# Residential Aged Care Pharmacist: An Australian Pilot Trial Exploring the Impact on Quality Use of Medicines Indicators

**DOI:** 10.3390/medicines7040020

**Published:** 2020-04-20

**Authors:** Nicole McDerby, Sam Kosari, Kasia Bail, Alison Shield, Gregory Peterson, Mark Naunton

**Affiliations:** 1 Discipline of Pharmacy, Faculty of Health, University of Canberra, Bruce ACT 2617, Australia; sam.kosari@canberra.edu.au (S.K.); alison.shield@canberra.edu.au (A.S.); g.peterson@utas.edu.au (G.P.); mark.naunton@canberra.edu.au (M.N.); 2 Discipline of Nursing, Faculty of Health, University of Canberra, Bruce ACT 2617, Australia; Kasia.Bail@canberra.edu.au; 3 Discipline of Pharmacy, Faculty of Health, University of Tasmania, Hobart TAS 7000, Australia

**Keywords:** aged care, drug burden index, hospitalisation, medication, nursing home, pharmacist, polypharmacy

## Abstract

**Background:** This pilot study aimed to assess whether an on-site pharmacist could influence indicators of quality use of medicines in residential aged care. **Methods:** A pharmacist was embedded in a residential aged care home for six months. A similar control site received usual care. Polypharmacy, drug burden index, antipsychotic and benzodiazepine use, hospital admission rates and length of stay, and emergency department presentation rates were outcomes used to indicate medication use quality. Data were extracted from participating resident health records. **Results:** Fifty-eight residents at the study site and 39 residents at the control site were included in the analysis. There was a reduction in the proportion of residents at the study site who had at least one hospital admission at follow-up (28% to 12%, *p* < 0.01), but no significant difference in other outcomes. **Conclusions:** This pilot study suggests that a residential care pharmacist may positively influence indicators of medication use quality in aged care; however, further research is needed to expand on these findings.

## 1. Introduction

Prescribing in older adults is complicated by multimorbidity, polypharmacy, age-related physiological changes, which alter drug pharmacokinetics and pharmacodynamics, and the involvement of multiple healthcare providers [1,2,3]. Up to 91% of individuals in the residential aged care home (RACH) setting are prescribed more than five concomitant medications and up to 74% of residents take more than nine medications [4]. Polypharmacy is implicated in an increased risk of exposure to a potentially inappropriate medication [1], and is associated with increased risk of drug interactions, falls, delirium and hospitalisation [5].

Specific medication classes are also associated with increased risk of harm in older adults. These include antipsychotics, benzodiazepines, and medications with anticholinergic effects [6]. The drug burden index (DBI) is a validated measure used to evaluate exposure to medications with anticholinergic or sedative effects, which may be used to estimate risk of potential medication-related harm [7]. Higher DBI in older adults is associated with adverse outcomes including increased frailty, worsening cognitive and functional impairment, falls, and hospital admissions [8,9,10]. Despite the established risks associated with the use of these medications in the RACH population [11], potentially inappropriate use remains an ongoing challenge [12,13].

Quality use of medicines is common nomenclature in Australia to describe best-possible medication use to maximise treatment benefit and minimise associated harm [14]. Discontinuing unnecessary or potentially inappropriate medications in people with limited life expectancy has been of growing interest. Identifying successful strategies in RACH is particularly important due to the prevalence of life-limiting diseases, such as advanced dementia [15]. Previous studies have reported interventions inclusive of pharmacist-led services in reducing resident exposure and usage of anticholinergic, antipsychotic and sedative medications [16,17]. However, no Australian studies have evaluated the effect of a pharmacist embedded as part of the on-site RACH care team on potentially inappropriate medication use.

The aim of this pilot study was to assess whether a residential care pharmacist (RCP) could influence indicators of quality use of medicines, through limited-efficacy testing [18]. The outcomes used as indicators were polypharmacy; DBI; antipsychotic and benzodiazepine use; hospital admission rates and length of stay; and emergency department (ED) presentation rates.

## 2. Materials and Methods

### 2.1. Setting

This study was conducted in two RACH located within the Australian Capital Territory (ACT), as part of a nonrandomised, controlled pilot trial [19]. Both sites belonged to the same organisation, operated under similar procedural guidelines and workplace cultures, with equal access to external health professionals, including general practitioners (GPs) and nurse practitioners. Nursing and care staff ratios were similar, with 70 nursing and care staff at the 104-bed study site and 80 staff at the 100-bed control site. The study site was nominated by the funding organisation. This study was approved by the University of Canberra Human Research Ethics Committee (HREC 16-244).

### 2.2. Participants

All residents at both RACH were eligible to participate in the study if they or their enduring power of attorney provided written consent prior to enrolment in the study. The RCP consented to participate and was purposefully recruited as the pharmacist already performing medication management reviews at both RACH on a visitational basis. The RCP was employed part-time (0.4 full time equivalent, two consecutive days per week) for six months at the study site. The RCP collaborated with the care team and documented all their activities performed for the study. These activities included both organisation-oriented quality improvement initiatives and resident-oriented clinical interventions.

### 2.3. Data Collection

Demographic and clinical information (age, gender, length of RACH admission, medical diagnoses) and medication usage data (drugs, doses, frequency of administration) for each resident from both RACH were recorded before and at the conclusion of the study period. All medications and diagnoses were coded as per the Anatomical Therapeutic Chemical (ATC) classification system [20] and International Classification of Disease (11th edition) [21], respectively. Antipsychotics included in the analysis were drugs indexed under ATC code N05A, excluding N05AN (lithium) and N05AB04 (prochlorperazine), as these drugs are not used to manage behavioural and psychological symptoms of dementia [12]. All regular daily dosages of antipsychotics and benzodiazepines were converted into chlorpromazine and diazepam daily dose equivalents, respectively [12]. Mean chlorpromazine and diazepam daily dose equivalents at baseline and immediately following the study period were used to assess trends in antipsychotic and benzodiazepine usage.

Total DBI for residents at baseline and post-RCP at both sites were calculated in accordance with validated methodology [8,22]. Medications and doses for the calculation of DBI were determined using the list published by Byrne et al. [23]. All vitamin supplements, topical medications, and medications taken as needed were excluded from the DBI analyses. Mean DBI scores at baseline and follow-up were used to assess trends in resident drug burden. DBI scores included antipsychotics and benzodiazepines due to the sedating nature of these medicines, in addition to the individual usage rates of these medicines outlined above.

Data on hospital admissions, including length of stay (counted as nights spent at hospital) and ED presentations were collected for all consenting residents for the six months preceding the study period, and the six months during the intervention.

### 2.4. Analysis

Descriptive statistics were used to summarise variables. Nonparametric tests were used to measure differences between outcome variables due to the non-normality of data distribution, and *p*-values of <0.05 were considered statistically significant. All statistical analyses were conducted using SPSS Statistics version 25.0 (IBM, Armonk, NY, USA).

## 3. Results

Seventy-four residents were recruited from the intervention site (71% of 104 residents) and 43 participants from the control site (43% of 100 residents). Fourteen residents at the study site and four residents at the control site were deceased at follow-up, and two residents at the study site did not have an accessible medication profile at both time points. Follow-up data were available for 58 residents at the study site and 39 residents at the control site. The characteristics of these participating residents are summarised in Table 1. Some residents at both sites were recruited to the study on admission to the RACH after the intervention period had commenced, and therefore had a duration of aged care admission less than six months. There were no significant differences in participant characteristics between the two sites. 

The RCP documented a total of 284 system-level and resident-level activities aimed at improving the quality use of medicines at the study site during the study period. Findings relating to polypharmacy, DBI, hospital admissions, and ED presentations are summarised in Table 2. Polypharmacy was identified at both RACH and at both time points. There was no difference in the median number of regular medications used per resident between time points or between the study and control sites at follow-up. There was also no difference in median DBI scores at either RACH post-study.

The proportion of residents with at least one hospitalisation in the preceding six months, while not significantly different between groups at baseline, had reduced significantly at follow-up at the study site (*p* < 0.01) but not at the control site (*p* = 0.50). Infections, including urinary tract infection and pneumonia, and falls were the most common causes of hospital admission at both RACH. Falls were implicated as the primary reason for hospital admission for six residents pre-study and one resident post-study at the study site, and four and two residents at the control site pre- and post-study, respectively. There was no difference in the number of residents presenting to ED between baseline and follow-up.

Antipsychotic and benzodiazepine results are summarised in Table 3. There were no significant differences observed in any of the outcomes relating to antipsychotic and benzodiazepine use.

## 4. Discussion

The results of this pilot study suggest that embedding an on-site pharmacist into RACH may influence some indicators of medication use quality, based on positive trends observed in hospital admission rates. Improvements in some of the other indicators may have been difficult to demonstrate for several reasons. In particular, it should be noted that the baseline quality use of medicine indicators were relatively sound at both RACH included in this study. For instance, there were already very low baseline rates of antipsychotic and benzodiazepine use compared to rates of approximately 20% reported elsewhere [12,24]. Therefore, there may have been insufficient scope for improvement in the quality use of medicines outcomes chosen in the study population [25].

The short study period of six months may have been an insufficient timeframe to assess the clinical impact of the pharmacist’s activities using the outcomes reported here. Balancing treatment efficacy and safety is complex in older, multimorbid adults [26], and identifying cause and clinical effect relationships between interventions and outcomes, such as hospital admissions and falls, is challenging in the RACH population. A key focus of the RCP was to conduct activities, such as quality improvement activities and staff education, directed towards implementing system-level change for improved medication safety [27].

Although polypharmacy can contribute to medication-related harm in RACH, it is not always inappropriate given the multimorbidity of this population, who may require long-term management of numerous chronic diseases. Observed proportions of residents prescribed more than five medications at each site were similar to rates of up to 91% reported elsewhere [4].

The quality use of medicines in RACH is influenced by numerous factors that contribute to the ‘prescribing culture’ within the organisation [13,28] These factors include staffing levels, workload and skill mix, managerial expectations and collaboration between visiting health professionals [13,28]. These factors extend beyond the control of interventions targeted at improving clinical appropriateness of medication use for individual residents. Interventions will likely yield greater success in improving quality use of medicines, and sustaining that improvement, if factors influencing prescribing culture and thereby the quality use of medicines within the organisation are addressed [13]. Additionally, proactive discussion between health care professionals, residents and family members is required to individualise deprescribing processes [15].

This study was limited by the small sample size available for inclusion at follow-up. Participation rates were approximately 50% at each included RACH; therefore, the included residents may not have been representative of the overall population. Due to funding constraints, this study was limited by the part-time nature of the RCP role, who was only on-site two days per week. This restricted the capacity of the pharmacist to prioritise clinical interventions towards individual residents who did not have high priority medication-related issues, and limited opportunities for synchronous discussion with GPs during visits to the RACH to facilitate timely problem resolution.

## 5. Conclusions

This pilot study has demonstrated that embedding an on-site pharmacist in RACH is feasible and may positively influence medication use quality indicators. However, further research with larger study populations across multiple sites is required to evaluate the effects of on-site pharmacists in improving the quality use of medicines for RACH residents.

## Figures and Tables

**Table 1:** Participant characteristics.

Characteristic	Study Site (n = 58)	Control Site (n = 39)	*p*-Value
Median age, years (IQR)	87.5 (82–91)	88 (82–91)	0.89 ^a^
Female gender, number (%)	38 (65.5)	22 (56.4)	0.40 ^b^
Median duration of aged care admission to end of intervention period, months (IQR)	22 (12–45.3)	15 (9–27)	0.14 ^a^
Documented dementia diagnosis, number (%)	26 (44.8)	23 (58.9)	0.22 ^b^
Median number of medical conditions per resident (IQR)	7 (5–9)	7 (6–9)	0.80 ^a^
Median Charlson Comorbidity Index (IQR)	6 (5–7)	5 (5–6)	0.46 ^a^

IQR, interquartile range; ^a^ Mann-Whitney U test; ^b^ Fisher’s exact test.

**Table 2:** Summary of use of medicines quality indicators.

Medicines Use Quality Indicator	Study Site (n = 58)		Difference (*p*-Value)	Control Site (n = 39)		*p*-Value
	Baseline	Follow-up		Baseline	Follow-up	
Number of residents prescribed >5 regular medications (%)	50 (86)	47 (81)	0.25 ^a^	30 (77)	30 (77)	1.00 ^a^
Median number of regular medications per resident (IQR)	9 (6–12.3)	9 (6–13)	0.90 ^b^	7 (6–10)	8 (6–10)	0.57 ^b^
Number of residents prescribed at least one regular anticholinergic or sedative medication (%)	43 (74)	46 (79)	0.25 ^a^	26 (67)	29 (74)	0.25 ^a^
Median DBI (IQR)	0.7 (0–1.3)	0.7 (0.3–1.3)	0.48 ^b^	0.5 (0–0.8)	0.5 (0–0.8)	0.13 ^b^
Number of residents who had at least one admission to hospital (%)	16 (28)	7 (12)	<0.01 ^a^	7 (18)	5 (13)	0.50 ^a^
Median length of hospital admission (IQR)	13.5 (3–28) (n = 16)	7 (3–12) (n = 7)	0.20 ^b^	11 (4–16) (n = 7)	8 (3.5–13.5) (n = 5)	0.50 ^b^
Number of residents who had at least one ED presentation (%)	10 (17)	13 (22)	0.25 ^a^	7 (18)	8 (21)	1.00 ^a^

DBI, drug burden index; ED, emergency department; IQR, interquartile range ^a^ McNemar test; ^b^ Wilcoxon signed rank test.

**Table 3:** Summary of antipsychotic and benzodiazepine use.

Usage by Drug Class	Baseline	Follow-up	*p*-Value
**Antipsychotics**			
**Number of residents prescribed a regularly-dosed antipsychotic**			
Study site, n = 58 (%)	7 (12)	6 (10)	1.00 ^a^
Control site, n = 39 (%)	5 (13)	4 (10)	1.00 ^a^
**Number of residents prescribed a ‘when required’ antipsychotic**			
Study site, n = 58 (%)	2 (3)	2 (3)	1.00 ^a^
Control site, n = 39 (%)	2 (5)	2 (5)	1.00 ^a^
**Median daily chlorpromazine dose equivalence (mg)**			
Study site (IQR)	50 (25–100) (n = 7)	25 (16.7–66.7) (n = 6)	0.46 ^b^
Control site (IQR)	50 (12.5–125.0) (n = 5)	25 (6.3–62.5) (n = 4)	0.14 ^b^
**Benzodiazepines**			
**Number of residents prescribed a regularly-dosed benzodiazepine**			
Study site, n = 58 (%)	11 (19)	10 (17)	1.00 ^a^
Control site, n = 39 (%)	1 (3)	2 (5)	1.00 ^a^
**Number of residents prescribed a ‘when required’ benzodiazepine**			
Study site, n = 58 (%)	5 (9)	6 (10)	1.00 ^a^
Control site, n = 39 (%)	2 (5)	4 (10)	0.50 ^a^
**Median daily diazepam dose equivalence (mg)**			
Study site (IQR)	5.0 (5–10) (n = 11)	7.5 (5–10) (n = 10)	0.41 ^b^
Control site (IQR)	2.5 (0–2.5) (n = 1)	5.0 (5–5) (n = 2)	0.32 ^b^

IQR, interquartile range; ^a^ McNemar Test; ^b^ Wilcoxon signed rank test.

## References

[1] Morin L., Laroche M.-L., Texier G., Johnell K. (2016). Prevalence of potentially inappropriate medication use in older adults living in nursing homes: A systematic review. J. Am. Med. Dir. Assoc..

[2] Clyne B., Cooper J.A., Hughes C.M., Fahey T., Smith S.M. (2016). ‘Potentially inappropriate or specifically appropriate?’ Qualitative evaluation of general practitioners views on prescribing, polypharmacy and potentially inappropriate prescribing in older people. BMC Fam. Pract..

[3] Alldred D.P., Kennedy M.-C., Hughes C., Chen T.F., Miller P. (2016). Interventions to optimise prescribing for older people in care homes. Cochrane Database Syst. Rev..

[4] Jokanovic N., Tan E.C.K., Dooley M.J., Kirkpatrick C.M., Bell J.S. (2015). Prevalence and factors associated with polypharmacy in long-term care facilities: A systematic review. J. Am. Med. Dir. Assoc..

[5] Jokanovic N., Wang K.N., Dooley M.J., Lalic S., Tan E.C.K., Kirkpatrick C.M., Bell J.S. (2016). Prioritizing interventions to manage polypharmacy in Australian aged care facilities. Res. Soc. Adm. Pharm..

[6] Davies E.A., O’Mahony M.S. (2015). Adverse drug reactions in special populations—The elderly. Br. J. Clin. Pharmacol..

[7] Hilmer S.N., Mager D.E., Simonsick E.M., Cao Y., Ling S.M., Windham B.G., Harris T.B., Hanlon J.T., Rubin S.M., Shorr R.I. (2007). A Drug Burden Index to Define the Functional Burden of Medications in Older People. Arch. Intern. Med..

[8] Hilmer S.N. (2018). Calculating and using the drug burden index score in research and practice. Expert Rev. Clin. Pharmacol..

[9] Kouladjian L., Gnjidic D., Chen T.F., Mangoni A.A., Hilmer S.N. (2014). Drug Burden Index in older adults: Theoretical and practical issues. Clin. Interv. Aging.

[10] Wouters H., van der Meer H., Taxis K. (2017). Quantification of anticholinergic and sedative drug load with the Drug Burden Index: A review of outcomes and methodological quality of studies. Eur. J. Clin. Pharmacol..

[11] Maust D.T., Kim H.M., Seyfried L.S., Chiang C., Kavanagh J., Schneider L.S., Kales H.C. (2015). Antipsychotics, other psychotropics, and the risk of death in patients with dementia: Number needed to harm. JAMA Psychiatry.

[12] Westbury J.L., Gee P., Ling T., Brown D.T., Franks K.H., Bindoff I., Peterson G.M. (2018). RedUSe: Reducing antipsychotic and benzodiazepine prescribing in residential aged care facilities. Med. J. Aust..

[13] Aerts L., Cations M., Harrison F., Jessop T., Shell A., Chenoweth L., Brodaty H. Why deprescribing antipsychotics in older people with dementia in long-term care is not always successful: Insights from the HALT study. Int. J. Geriatr. Psychiatry.

[14] Sluggett J.K., Ilomäki J., Seaman K.L., Corlis M., Bell J.S. (2017). Medication management policy, practice and research in Australian residential aged care: Current and future directions. Pharmacol. Res..

[15] Kalogianis M.J., Wimmer B.C., Turner J.P., Tan E.C.K., Emery T., Robson L., Reeve E., Hilmer S.N., Bell J.S. (2016). Are residents of aged care facilities willing to have their medications deprescribed?. Res. Soc. Adm. Pharm..

[16] McLarin P.E., Peterson G.M., Curtain C.M., Nishtala P.S., Hannan P.J., Castelino R.L. (2016). Impact of residential medication management reviews on anticholinergic burden in aged care residents. Curr. Med. Res. Opin..

[17] Westbury J., Jackson S., Gee P., Peterson G. (2010). An effective approach to decrease antipsychotic and benzodiazepine use in nursing homes: The RedUSe project. Int. Psychogeriatr..

[18] Bowen D.J., Kreuter M., Spring B., Cofta-Woerpel L., Linnan L., Weiner D., Bakken S., Kaplan C.P., Squiers L., Fabrizio C. (2009). How we design feasibility studies. Am. J. Prev. Med..

[19] McDerby N., Naunton M., Shield A., Bail K., Kosari S. (2018). Feasibility of integrating residential care pharmacists into aged care homes to improve quality use of medicines: Study protocol for a non-randomised controlled pilot trial. Int. J. Environ. Res. Public Health.

[20] World Health Organization Collaborating Centre for Drug Statistics Methodology ATC/DDD index [Internet]. https://www.whocc.no/atc_ddd_index/.

[21] World Health Organization (2018). International classification of diseases for mortality and morbidity statistics (11th Revision) [Internet]. https://icd.who.int/browse11/l-m/en.

[22] Crotty M., Rowett D., Spurling L., Giles L.C., Phillips P.A. (2004). Does the addition of a pharmacist transition coordinator improve evidence-based medication management and health outcomes in older adults moving from the hospital to a long-term care facility? Results of a randomized, controlled trial. Am. J. Geriatr. Pharmacother..

[23] Byrne C.J., Walsh C., Cahir C., Ryan C., Williams D.J., Bennett K. (2018). Anticholinergic and sedative drug burden in community-dwelling older people: A national database study. BMJ Open.

[24] Westbury J., Beld K., Jackson S., Peterson G. (2010). Review of psychotropic medication in Tasmanian residential aged care facilities. Australas J. Ageing.

[25] Muth C., Uhlmann L., Haefeli W.E., Rochon J., van den Akker M., Perera R., Güthlin C., Beyer M., Oswald F., Valderas J.M. (2018). Effectiveness of a complex intervention on Prioritising Multimedication in Multimorbidity (PRIMUM) in primary care: Results of a pragmatic cluster randomised controlled trial. BMJ Open.

[26] Hernandez M.H., Mestres C., Modamio P., Junyent J., Costa-Tutusaus L., Lastra C.F., Mariño E.L. (2019). Adverse Drug Events in Patients with Dementia and Neuropsychiatric/Behavioral, and Psychological Symptoms, a One-Year Prospective Study. Int. J. Environ. Res. Public Health.

[27] McDerby N., Kosari S., Bail K., Shield A., Peterson G., Naunton M. (2019). The effect of a residential care pharmacist on medication administration practices in aged care: A controlled trial. J. Clin. Pharm. Ther..

[28] Sawan M., Jeon Y.H., Fois R.A., Chen T.F. (2017). Exploring the link between organizational climate and the use of psychotropic medicines in nursing homes: A qualitative study. Res. Soc. Adm. Pharm..

